# The Effect of Muscle Cramps During Hemodialysis on Quality of Life and Habitual Physical Activity

**DOI:** 10.3390/medicina60122075

**Published:** 2024-12-18

**Authors:** Gabriela Kot, Agata Wróbel, Kasper Kuna, Agnieszka Makówka, Michał Nowicki

**Affiliations:** 1 Faculty of Medicine, Medical University of Lodz, Pomorska 251, 92-213 Lodz, Poland; gabriela.ewa.kot@outlook.com (G.K.);; 2 Department of Nephrology, Hypertension, Transplantation and Internal Medicine, Central University Hospital, Medical University of Lodz, Pomorska 251, 92-213 Lodz, Poland

**Keywords:** hemodialysis, IPAQ, muscle spasms, SF-36, quality of life

## Abstract

*Background and Objectives*: This study aimed to evaluate the association between QoL, self-assessed physical activity, and the presence and severity of muscle spasms in chronic hemodialysis patients. Patients undergoing regular in-center hemodialysis (HD) have much lower quality of life (QoL) than healthy individuals. However, there is limited knowledge about the impact of specific common complications of hemodialysis, particularly muscle spasms on the overall well-being of patients. *Materials and Methods*: In this prospective, single-center study, 67 chronic HD patients were surveyed regarding the prevalence of muscle spasms using a validated 9-multiple-choice-question survey, alongside the Physical Activity Questionnaire (IPAQ) and The Short Form (36) Health Survey (SF-36). Based on the muscle spasms survey answers, patients were divided into two subgroups: with (*n* = 39) and without muscle spasms (*n* = 28). *Results*: The findings revealed that patients with muscle spasms had a higher body mass index (BMI) (*p* = 0.005), a shorter dialysis vintage (*p* = 0.063), and significantly longer sitting times (*p* = 0.017). Multivariate analysis identified BMI (*p* = 0.034), sitting time (*p* = 0.009), physical functioning scores (*p* = 0.032), and dialysis vintage (*p* = 0.040) as significant predictors of muscle spasms. *Conclusions:* This study concluded that muscle spasms are associated with lower QoL among HD patients. The contributing factors to this dependance are BMI, dialysis vintage, physical functioning, and sitting time.

## 1. Introduction

Hemodialysis (HD) is associated with numerous side effects that can result from both dialysis process itself and disease progression. These adverse symptoms mainly include fatigue, muscle spasms, electrolyte imbalances, and hypotension, which togheter lead to a decrease in a patient’s quality of life (QoL) [1]. However, in most cases, HD remains one of the few treatments options for patients with end-stage renal disease (ESRD), which offers the management of fluid balance and effectively removes waste products [2]. As a result, reducing and understanding the impact of HD side effects has become one of the clinical priorities.

Muscle spasms are a common complication during hemodialysis (HD), affecting approximately 5–20% of chronic hemodialysis patients, more prevalent within the first months of HD treatment [3]. These painful cramps typically occur in the lower extremities and may be severe enough to disrupt HD treatment. They typically arise in the second half of HD session, most probably due to increasing skeletal muscle ischemia induced by maximal water removal through ultrafiltration (UF). Although muscle spasms can spontaneously resolve within minutes after the completion of a dialysis session, they may sometimes persist for several hours post-dialysis. Muscle spasms are one of the most significant causes of non-adherence to the recommended HD treatment [1].

A key measure of the effectiveness of HD and the well-being of chronic hemodialysis patients’ treatment is the quality of life (QoL) [4,5]. Overall QoL is a sum of several aspects of a patient’s well-being, like physical activity, mental health, and social aspects of life. In clinical medicine, QoL assessment is commonly used to evaluate the effects of different diseases and their treatment on patients’ well-being [6]. In addition to end-stage kidney disease dialysis, treatment itself has a profound impact on the QoL of patients, primarily due to physical burdens, frequent associated symptoms, side effects, and psychological and social impacts [7,8]. The main risk factors that lower QoL among HD patients are common co-morbidities, older age, long dialysis sessions, and malnutrition [9,10,11]. Various instruments and questionnaires were developed to measure the quality of life in the adult population [12,13].

The most widely used and best-validated survey, SF-36 Health Survey, is a versatile, short-form health assessment tool consisting of 36 questions. It is designed as a general health status measure rather than being specific to any particular age, disease, or underlaying disease treatment. The SF-36 evaluates patient-reported responses across eight health domains, i.e., physical functioning, role physical, bodily pain, general health perception, vitality, social functioning, role emotional, and mental health [14]. It has been translated and culturally adapted to many countries, including Poland [15].

The International Physical Activity Questionnaire (IPAQ) is a standardized self-reported measurement tool. This questionnaire is used to measure physical activity and sedentary behavior in adults aged 15 to 69. It is available in both short (7 questions) and long (27 questions) forms, evaluating the frequency and duration of various activities. The IPAQ is validated for diverse socio-economic and cultural settings [16].

Despite numerous studies addressing muscle spasms in dialysis patients, the extent to which muscle spasms impact the quality of life and activity levels in hemodialysis patients remains unclear.

Objective: This study evaluated the association between QoL, self-assessed physical activity, and the presence and severity of muscle spasms in chronic hemodialysis patients.

## 2. Materials and Methods

### 2.1. Participants

This prospective, single-center study included 90 patients undergoing chronic hemodialysis treatment 3 times a week for at least 6 months. Each patient had to have a constant “dry weight” for at least the previous 4 weeks. An episode of muscle spasm was defined as the occurrence of one or more episodes of painful, involuntary muscle contractions lasting longer than one minute, occurring either during or shortly after hemodialysis, and within the preceding two weeks. Medical records were collected from all patients; focusing on the exclusion criteria, 67 out of 90 patients were ultimately included.

Each participant of the final study cohort (*n* = 67) was assessed for demographics (age range 27–69, mean 60 ± 8.8 years) and HD-specific variables. The gender ratio included an approximately equal representation of male and female patients (35F; 32M), with slight deviations reflecting the demographics of the local HD population. The patients completed a validated questionnaire consisting of nine multiple-choice questions about the prevalence of muscle spasms. Based on the survey responses, the patients were divided into two groups, i.e., experiencing muscle spasms (*n* = 39) and without muscle spasms (*n* = 28).

### 2.2. Dialysis Process Parameters

All patients in this study underwent chronic hemodialysis using biocompatible membranes and bicarbonate-based dialysate. These parameters were standardized across the cohort to ensure uniform dialysis protocols. The adequacy of dialysis was confirmed with a minimum Kt/V of 1.2 for all patients, meeting clinical guideline standards. The average dialysis procedure time among patients was 215 min (mostly 210 min, minimum 135 min and max 285 min). The mean interdialytic weight gain was 2.86 ± 1.1 kg. These parameters were comparable between the spasm and no-spasm groups, suggesting no significant differences in fluid management between the two subgroups. Sodium profiling was not routinely measured, and information regarding its use was not available for analysis.

### 2.3. Exclusion Criteria

Exclusion criteria were as follows: malignancy, severe heart insufficiency (New York Heart Association (NYHA) Functional Classification stage III or IV), liver failure, documented muscular diseases, recent bone fractures, inability to walk, mental illnesses, treatment-resistant hypertension, and significantly reduced physical activity due to diseases of the nervous, muscular, and skeletal systems.

### 2.4. Study Procedures

Each patient was asked to complete the Short Form 36 (SF-36) questionnaire and the International Physical Activity Questionnaire (IPAQ) before HD sessions to capture baseline physical and mental health. The SF-36 survey covers 8 health domains: physical functioning, bodily pain, social functioning, and others. The IPAQ was used in its long form to assess the frequency, duration, and intensity of physical activity, expressed as MET scores. The evaluation was conducted by an interviewer whenever a patient had difficulty understanding the items. The evaluation also included the basic parameters of dialysis, such as time of dialysis, dialysis vintage, frequency, as well as key background factors as age, body mass, body mass index (BMI), and blood pressure (BP), obtained from medical records or direct physical examination.

### 2.5. Assessment of Physical Activity

Participants were asked to complete the IPAQ (long form); then, based on responses, the metabolic equivalents (MET) were calculated. Oxygen consumption at rest of approximately 200–250 mL/minute, or 3.5 mL/kg/minute, was defined as one MET [16]. Total METs were calculated as the sum of METs assigned to each activity per week in the IPAQ multiplied by the activity index as appropriate:

- 3.3 × MET for walking/per week
- 4.0 × MET for moderate activity/per week
- 3.0 × MET for moderate activity related to work at home/per week
- 8.0 × MET for vigorous activity/per week
- 5.5 × MET for vigorous activity related to work around home/per week
- 6.0 × MET for cycling as a form of transport/per week [16].

### 2.6. Statistical Analysis

Patient characteristics were depicted using standard descriptive statistics methods for continuous and categorical variables to summarize demographic and clinical characteristics across groups (patients with and without muscle spasms). Then, groups were compared with X^2^ test or *t*-test for categorical and numerical variables, respectively. Before conducting the *t*-tests, data were assessed for normality using the Shapiro–Wilk test. In cases where the assumptions of normality were violated, the Mann–Whitney U test was considered. Univariate analysis was conducted to determine the association of muscle spasms with QoL, physical activity, and every score of the SF-36 and IPAQ. Each variable was tested individually to determine its odds ratio and confidence interval. Following univariate analysis, variables that showed statistical significance were included in a multivariate logistic regression, which was performed, with the occurrence of muscle spasms as the dependent variable. To account for potential interactions between variables, we analyzed the correlation matrix, including a potential interaction term for BM and BMI (BM*BMI). No significant multicollinearity was observed, and BMI was chosen over BM for the multivariate analysis due to its stronger association with muscle cramps. In addition, a Receiver Operating Characteristic (ROC) curve was drawn to evaluate the model’s predictive performance, as well as the determination of specificity and sensitivity. Area Under the Curve (AUC) was calculated as an indicator of the model’s accuracy, while Akaike Information Criterion (AICc) was used to assess the model’s quality and to help in model selection.

Statistical analysis was performed using Statistica software (13.1 version, TIBCO Software Inc., Palo Alto, CA, USA). All tests were two-sided, and *p*-value < 0.05 was considered statistically significant.

### 2.7. Ethical Considerations

This study was approved by the Bioethics Committee of the Medical University of Lodz, number RNN/163/20/KE, 16 June 2020. This study was conducted in accordance with the Declaration of Helsinki. A written informed consent form was received from all participants prior to the study.

## 3. Results

In total, 67 out of 90 originally enrolled patients underwent all study procedures. The baseline characteristics of the study population with a comparison of the group with and without muscle spasms are presented in Table 1. Two groups significantly differed only with respect to body mass and BMI. Patients experiencing muscle spasms demonstrated significantly higher BMI compared to those without spasms (28.2 ± 5.6 vs. 25.0 ± 5.1; *p* = 0.004).

Table 2 shows the results of the univariate analysis of the patient baseline characteristics. BMI was associated with a higher risk of muscle spasms (OR 1.132 95% Cl 1.016–1.260, *p* = 0.04). Moreover, a shorter dialysis vintage was associated with a decreased risk of muscle spasms (OR 0.999 95% Cl 0.999–1.000, *p* = 0.03).

The scoring results of the SF-36 questionnaire in the entire study population and in groups with and without muscle spasms are presented in Table 3. No statistically significant differences were found between the scores.

IPAQ scores (Table 4) showed that the patients who reported muscle spasms had numerically lower physical activity compared to the patients without spasms; however, the difference was not statistically significant (48.8% vs. 25.0%, *p* = 0.330). Similarly, the Chi-Square test for the distribution of physical activity levels across the groups showed no statistical significance (*p* = 0.1434). The median METs for patients with muscle spasms was lower (METs between 600 and 1500; median category) than in the group without muscle spasms (METs > 1500; high category). However, this difference was not statistically significant (*p* = 0.205); thus, this parameter should not be considered as a predictor. In contrast, the difference in the mean sitting time between the groups was statistically significant (513.3 ± 227.4 vs. 336.4 ± 194.8; *p* = 0.017).

### METs—Metabolic Equivalents

Univariate logistic regression analysis of QoL and self-assessed physical activity (Table 5) showed that the physical functioning score was lower among patients with than without muscle spasms (OR 0.984 95% Cl 0.966–1.003, *p* = 0.016). Sitting time was higher among patients suffering from muscle spasms versus the patients who did not report muscle spasms (OR 1.003 95% Cl 1.001–1.006, *p* = 0.011). Other scores did not show a statistically significant difference, and thus these variables are noted as trends rather than definitive predictors.

Multivariate logistic regression analysis was performed, including the parameters that were significant predictors in univariate analysis—BMI, sitting time, dialysis vintage, and physical functioning. Table 6 presents the results of the multivariate analysis. To avoid errors due to multicollinearity, the correlation matrix between variables was analyzed.

The logistic regression analysis showed that a higher risk of muscle spasms was associated with increased BMI (*p* = 0.034) and extended periods of sitting (*p* = 0.009). Higher scores in physical functioning category were associated with a decreased risk of muscle spasms (*p* = 0.032). Longer time on dialysis treatment was associated with a lower risk of muscle spasms (*p* = 0.040). That may indicate that patients with longer HD treatment histories and higher physical activity may experience fewer spasms.

**Figure 1:**
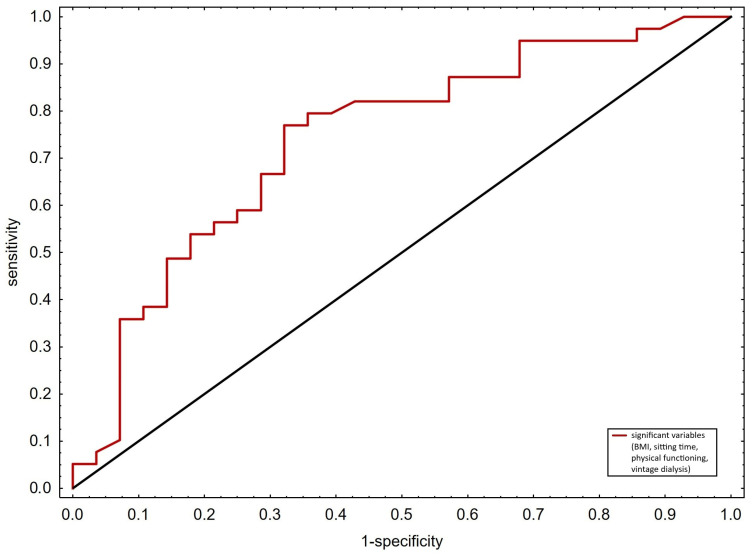
ROC curve. Sensitivity: 79.5%; specificity: 64.3%; AUC: 0.739; AICc value: 79.3.

Based on the results of multivariate logistic regression analysis, an ROC curve (Figure 1) was created to evaluate the model performance based on significant variables (BMI, dialysis vintage, sitting time and physical functioning). The analysis showed a sensitivity of 79.5%, specificity of 64.3%, an AUC of 0.739, and an AICc value of 79.3.

## 4. Discussion

To gain a better understanding of how muscle spasms impact patients QoL, this study evaluated the association between these two variables, also taking into consideration several background factors. The main finding of our study was that muscle spasms were significantly associated with lower QoL scores in only two areas, that is, physical functioning and time spent seated during the day. Additionally, muscle spasms were correlated with higher BMI and shorter dialysis vintage. Our model of ROC curve demonstrated high sensitivity and moderate specificity in predicting the presence of muscle spasms based on BMI, dialysis vintage, physical functioning, and sitting time.

The simplest and most cost-effective methods to access QoL scores in chronically ill patients is through self-reported questionnaires, such as the SF-36 and IPAQ, which werevalidated for numerous disease settings [17]. The SF-36 questionnaire was used in many studies examining the QoL of HD patients [18,19,20]. To date, the IPAQ has not been commonly used among HD patients, and only several studies attempted to validate its use [21,22]. These studies found that IPAQ is a reliable tool for accessing physical activity levels in this highly specific patient group. Additionally, the IPAQ offers a comprehensive evaluation of physical activity, making it a valuable asset for understanding its impact on QoL in HD patients.

A recent meta-analysis by Fletcher et al., where 449 studies on the QoL of patients with chronic kidney disease (CKD) showed overall decreased QoL in CKD patients, with hemodialysis patients experiencing the lowest QoL. Another study also revealed a significant symptoms burden but did not analyze the individual impact of each symptom separately [23]. Our study extended those observations by establishing associations between one domain of the SF-36 questionnaire and prevalence of muscle spasms. From our observations, we concluded that there is a particularly significant association between muscle spasms and physical functioning. To the best of our knowledge, our study highlighted, for the first time, that the frequent occurrence of muscle spasms not only negatively affects patients’ functioning during and shortly after hemodialysis but also significantly impairs everyday activities, leading to a further reduction in the overall quality of life.

We proved that the median physical activity level in HD patients is classified as moderate for those experiencing muscle spasms but high for those who do not experience them. This result contrasts with some other studies which measured physical activity in HD patients that typically indicated its highly decreased levels [24,25]. The study by Wong et al. found that none of the analyzed 70 HD subjects exhibited a high level of physical activity, whereas among our patients, more than 30% declared high physical activity in the IPAQ [26]. These differences may result from the exclusion criteria of our study, resulting in a high percentage of patients with lower number of comorbidities compared to other studies. Additionally, most of the previous studies used non-subjective methods for physical activity measurement, e.g., accelerometers and pedometers, whereas our study relied on self-assessment.

However, it is important to consider the possibility of reverse causality between physical activity and muscle spasms among HD patients. In some cases, muscle spasms may precede reduced physical activity. The pain and discomfort associated with cramps can discourage patients from engaging in physical activity [27]. Patients also may develop anxiety or fear related to the possibility of spasms occurring during exercise. It can lead to avoidance behavior, which may contribute to a sedentary lifestyle, leading to further muscle weakness, increasing the likelihood of future cramps, creating a cycle of dependancy [28,29]. It is difficult to assess which factor is the primary cause, as physical activity and muscle spasms influence each other in a positive feedback loop.

Another parameter examined in the IPAQ was time spent seated during the day, which was found to be significantly associated with the occurrence of muscle spasms. Musolino et al. found a significant association between prolonged sitting time in chronic dialysis patients and the deterioration of their mental well-being, which thereby led to a decline in their quality of life [30]. This finding aligned with a previous study that found that increased sedentary behavior negatively impacted mental health outcomes and QoL [31]. All this may suggest that a sedentary behavior could be associated with the occurrence of muscle spasms in HD patients, highlighting the importance of including both physical activity and sedentary behavior when analyzing the prevalence of muscle spasms.

Another interesting finding of our study was that muscle spasms more often occurred in patients with higher BMI. Ul-Haq et al. conducted a meta-analysis and found out that, not only physical, but also mental domains of QoL were lower in patients with obesity and comorbidities [32]. In another study that included 134 healthy individuals with abnormal BMI, a negative correlation between high BMI and QoL was also revealed [30]. Therefore, our results corroborated these findings, suggesting that muscle spasms may have a negative effect on the quality of life. We may conclude that since patients with a higher BMI more frequently experience cramps, there is a group of patients in which both factors overlap, further diminishing their quality of life. However, that does not mean that low BMI is desirable for dialysis patients, e.g., Yang et al. indicated that extremely low BMI is associated with increased mortality among HD patients [33]. It may suggest that there is a crucial balance to be obtained when considering patients with BMI outside the normal range and persistent muscle spasm.

Not unexpectedly, our study also confirmed that longer dialysis vintage had a negative effect on QoL [2,34,35,36,37,38]. This is affected by various factors such as mental fatigue of patients due to constant contact with healthcare services, continuous medical procedures, constantly deteriorating health, concerns about their own and their family’s future, inability to work, their financial situations, etc. [35,39,40]. Side effects of HD affect the majority of patients and are one of main reasons for reduced QoL [23,37,41]. Moreover, most of these factors emerge and intensify with the duration of HD therapy [37]. Interestingly, the muscle cramps we studied exhibited an opposite tendency. In our study, we demonstrated that the longer a patient undergoes dialysis therapy, the lower the probability of experiencing painful muscle spasms. Some authors speculated that the relationship between the duration of HD therapy and the occurrence of muscle cramps could be explained by the progressive sarcopenia [42,43,44]. As a result, muscle cramps may become less bothersome and eventually cease. However, Thijssen et al. indicated that many patients are malnourished at the beginning of dialysis therapy, which improves with the duration of HD [45]. Since higher BMI correlates with more frequent cramps, as we presented, they seem to exhibit the opposite tendency.

The etiology of muscle cramps in HD patients is complex and may be influenced by many factors contributing to the overall outcome of the treatment. One of the most common causes is dialysis-related hypotension, which is present in 15–30% of patients due to the inadequate cardiovascular response to decreased blood volume after dialysis, causing the hypoxia of the muscles [46]. Also, the electrolyte disturbance caused by ultrafiltration during hemodialysis can lead to the muscle cramps. Hypomagnesaemia has not been only associated with increased mortality but also linked to an increased risk of muscle cramps [47]. Electrolyte imbalance such as decreased level of sodium and potassium and elevated phosphorus level can cause muscle spasms [3]. Another important factor causing prolonged muscle contraction is the carnitine deficiency, which is caused by the removal of free carnitine during the hemodialysis process. The L-carnitine substitution in HD patients is recommended as the carnitine plays important in the transport of long-chain fatty acids to the mitochondrial matrix [48]. Along with carnitine deficiency, there is also a plasma alkalosis which could be either due to dialysate or contraction alkalosis caused by fluid loss after HD. It causes hypocalcemia by binding the calcium ions to the albumins and further releasing calcium ions from the sarcoplasmic reticulum, resulting in muscle spasms [49]. Researchers are aware of the above-mentioned and other risk factors not accessed in this study; this research, however, focused on patient-reported quality of life outcomes. There is a need for further research to adjust the above-mentioned factors.

The primary limitation of our study is the small sample size. Increasing the number of patients through a longer study duration or a multi-center approach would significantly enhance the reliability of the results. Additionally, this study included only subjective methods, such as self-reported questionaries. Future research would benefit from incorporating objective measures (e.g., 3D accelerometers), which could provide more accurate and reliable data. Another limitation of our study is the lack of continuous monitoring of blood pressure during dialysis. However, due to a limited population studied, we selected the patients very carefully to include a group that was as homogenous as possible in the case of chronic dialysis patients. None of the patients included in our study population had a history of either symptomatic hypotension or intradialytic hypotension, and we did not include patients with autonomic neuropathy. Therefore, it is unlikely that intradialytic decreases in blood pressure could have had any significant effect on the incidence of muscle spasms in our patients. Furthermore, while BMI was included as an indicator of general health, we recognize that other nutritional factors were not addressed. We acknowledge that these additional nutritional parameters could significantly enhance the understanding of QoL outcomes of HD patients. We acknowledge that the lack of data on the exact frequency and severity of muscle cramps is a limitation of this study. Future studies should consider collecting detailed data on the number of cramp episodes and stratifying patients into severity groups (e.g., mild, moderate, and severe) to better understand the relationship between cramp intensity and quality-of-life parameters. Previous studies assessing QoL using the SF-36 questionnaire mainly involved the comparison of hemodialyzed patients to healthy individuals or focused on symptoms that collectively impact quality of life [50,51,52,53]. Our study, unlike previous ones, investigated the relationship between QoL and muscle spasms in a population of chronic hemodialysis patients. We considered several different aspects of both mental and physical functioning, as well as other characteristics such as SBP, dialysis duration, and dialysis vintage.

Since we found that muscle cramps reduce QoL and they occur mainly at the beginning of HD therapy, we can conclude that in patients experiencing cramps, QoL is expected to decline faster than in those not experiencing them. Low QoL is associated with more frequent hospitalizations, reluctance to start or continue treatment, and increased mortality. Therefore, patients experiencing muscle cramps should be closely monitored for depressive disorders and other mood disturbances. Unfortunately, the pathogenesis of muscle spasms in dialysis patients is still not completely understood; therefore, its prophylaxis is not available. This leads to the conclusion that further studies investigating the pathogenesis and treatment of cramps in HD patients are warranted.

## 5. Conclusions

Our study underscored the significant negative impact of muscle spasms on the quality of life in hemodialysis patients, affecting physical and mental well-being. We found that the risk of muscle cramps increase with the amount of time spent sitting during the day and body mass index and negatively correlates with dialysis vintage. Additionally, muscle cramps may adversely affect physical activity among HD patients, which further diminishes their quality of life.

## Figures and Tables

**Table 1:** Overview of patient cohort.

Parameter	All Patients (*n* = 67)	With Muscle Spasms (*n* = 39)	Without Muscle Spasms (*n* = 28)	*p*-Value
Age [years] ± SD	60 ± 8.8	61.6 ± 7.1	57.9 ± 10.5	0.338
Body mass [kg] ± SD	78.4 ± 20.5	82.5 ± 19.2	72.8 ± 21.2	0.005
BMI [kg/m^2^] ± SD	26.9 ± 5.6	28.2 ± 5.6	25 ± 5.1	0.004
SBP [mmHg] ± SD	143.2 ± 24.4	144.5 ± 24.8	141.5 ± 24	0.617
Time of dialysis session [min] ± SD	215 ± 28.6	216.2 ± 29.9	213.6 ± 27.1	0.719
Dialysis vintage [Days] ± SD	993.7 ± 760.1	830.7 ± 692.7	1220.8 ± 803.2	0.063

SD—standard deviation, BMI—body mass index, SBP—systolic blood pressure.

**Table 2:** Univariable analysis of patient background factors.

Parameter	OR (95% Cl)	*p*-Value
Age	1.015 (0.984–1.047)	0.33
Body mass	1.026 (0.998–1.059)	0.04
BMI	1.132 (1.016–1.260)	0.01
SBP	1.005 (0.985–1.026)	0.61
Time of dialysis session	1.003 (0.986–1.021)	0.71
Dialysis vintage	0.999 (0.999–1.000)	0.03

OR—odds ratio, BMI—body mass index, SBP—systolic blood pressure.

**Table 3:** Comparison of QoL categories between groups with and without muscle spasms.

Score	All Patients (*n* = 67)	With Muscle Spasms (*n* = 39)	Without Muscle Spasms (*n* = 28)	*p*-Value *
Physical functioning	55.5 ± 27.4	50.8 ± 27.9	62 ± 25.8	0.112
Limitations due to physical health	37.7 ± 42.5	32 ± 41.3	45.5 ± 43.6	0.219
Limitations due to emotional problems	52.7 ± 45.4	49.6 ± 46.4	57.1 ± 44.3	0.496
Pain	60.6 ± 32.2	61 ± 34	60 ± 30	0.819
Emotional well-being	65.9 ± 19.1	65.6 ± 21.2	66.3 ± 16.3	0.804
Social Functioning	72.4 ± 27.8	70.8 ± 29.8	74.52 ± 25.1	0.794
Fatigue	45.2 ± 20.4	44.2 ± 20.9	46.6 ± 20.1	0.620
General Health	35.7 ± 12.5	35.9 ± 12.2	35.5 ± 13.2	0.484

* *p*-value represent the comparison between the presence or absence of muscle spasms.

**Table 4:** Comparison of results of the Internacional Physical Activity Questionnaire (IPAQ) between subgroups with and without muscle spasms.

	Physical Activity			Median Total METs-Min/Week	Mean Sitting Time [Min]
	Low	Moderate	High		
All patients (*n* = 67)	26 (38.8%)	20 (29.9%)	21 (31.3%)	953	451.9 ± 225.0
With muscle spasms (*n* = 39)	19 (48.8%)	10 (25.6%)	10 (25.6%)	724.5	513.3 ± 227.4
Without muscle spasms (*n* = 28)	7 (25.0%)	10 (35.7%)	11 (39.3%)	1699.5	336.4 ± 194.8
*p*-value *	0.1434			0.205	0.017

* *p*-values represent the comparison between the presence or absence of muscle spasms.

**Table 5:** Univariable logistic regression of SF-36 and IPAQ questionnaires answers between two group of patients: with and without muscle spasms.

Score	OR (95% Cl)	*p*-Value
Physical functioning	0.984 (0.966–1.003)	0.016
Limitations due to physical health	0.992 (0.981–1.004)	0.184
Limitations due to emotional problems	0.996 (0.986–1.007)	0.750
Pain	1.001 (0.986–1.016)	0.423
Emotional well-being	0.998 (0.973–1.024)	0.889
Social Functioning	0.995 (0.978–1.013)	0.133
Fatigue	0.994 (0.970–1.018)	0.196
General Health	1.002 (0.964–1.042)	0.859
Total METs	0.963 (0.912–1.012)	0.910
Time spent sitting during the day	1.003 (1.001–1.006)	0.011

OR—odds ratio, METs—metabolic equivalents.

**Table 6:** Results of the multivariate logistic regression analysis.

Variable	OR (95% Cl)	*p*-Value
Intercept	0.993 (0.003–2.969)	0.181
BMI	1.132 (1.009–1.270)	0.034
Dialysis vintage	0.999 (0.998–1.000)	0.040
Time spent sittingduring the day	1.004 (1.001–1.007)	0.009
Physical functioning	0.975 (0.953–0.998)	0.032

OR—odds ratio, BMI—body mass index.

## Data Availability

All data generated or analyzed during this study are included in this article. Further inquiries can be directed to the corresponding authors.
